# Demographic, Psychosocial, and Lifestyle-Related Characteristics of Forest Therapy Participants in Italy: A Multicenter Cross-Sectional Survey

**DOI:** 10.3390/healthcare11111627

**Published:** 2023-06-02

**Authors:** Michele Antonelli, Davide Donelli, Valentina Maggini, Eugenia Gallo, Vittorio Mascherini, Fabio Firenzuoli, Gioele Gavazzi, Federica Zabini, Emanuela Venturelli, Giovanni Margheritini, Ivana Bassi, Luca Iseppi, Francesco Meneguzzo

**Affiliations:** 1 Department of Public Health, AUSL-IRCCS di Reggio Emilia, Via Amendola 2, 42122 Reggio Emilia, Italy; 2 Division of Cardiology, Azienda Ospedaliero-Universitaria di Parma, Via Gramsci 14, 43126 Parma, Italy; donelli.davide@gmail.com; 3 CERFIT, Careggi University Hospital, Largo Brambilla 3, 50134 Florence, Italy; valentina.maggini@unifi.it (V.M.); eugenia.gallo@unifi.it (E.G.); vittorio.mascherini@gmail.com (V.M.); fabio.firenzuoli@unifi.it (F.F.); 4 Section of Psychology, Department of Neuroscience, Psychology, Drug Research and Child’s Health (NEUROFARBA), University of Florence, Via di San Salvi 12, 50135 Firenze, Italy; gioele.gavazzi@unifi.it; 5 Institute of Bioeconomy, National Research Council, Via Madonna del Piano 10, 50019 Sesto Fiorentino, Italy; federica.zabini@cnr.it (F.Z.); francesco.meneguzzo@cnr.it (F.M.); 6 Psychologist Group, Central Medical Commission, Italian Alpine Club, Via E. Petrella 19, 20124 Milano, Italy; emanuelaventurellipsicologa@gmail.com (E.V.); giomarghe@yahoo.com (G.M.); 7 Department of Agricultural, Food, Environmental and Animal Sciences, University of Udine, Via delle Scienze 206, 33100 Udine, Italy; ivana.bassi@uniud.it (I.B.); luca.iseppi@uniud.it (L.I.)

**Keywords:** forest bathing, forest therapy, public health, social sciences, psychology, preventive medicine, survey, Italian population

## Abstract

This research aims to provide a comprehensive overview of the key demographic, psychosocial, and lifestyle-related characteristics of forest therapy participants in Italy. A survey was conducted among 1070 adults who had engaged in standardized forest therapy experiences between June 2021 and October 2022. The findings indicate that most forest therapy participants in Italy share certain distinctive traits. They are primarily female, aged between 45 and 54 years, employed, and unmarried. Moreover, they possess a high level of education, predominantly reside in urban areas, demonstrate a strong environmental awareness, maintain a nature-oriented attitude, and typically exhibit moderate levels of trait anxiety. Additionally, they tend to be nonsmokers, possess a healthy BMI within the normal range, and consume an adequate quantity of fruits and vegetables on a daily basis. However, it should be noted that their male counterparts tend to be overweight and exhibit poorer dietary habits. Irrespective of gender, approximately 40% of forest therapy participants in Italy live with a chronic disease that requires daily medicinal treatment. Subsequent research should investigate whether these characteristics hold true in different countries. Furthermore, exploring the potential effectiveness of health-promoting interventions integrated with forest therapy sessions could prove beneficial in addressing these specific issues among forest therapy participants. By doing so, such interventions have the potential to contribute significantly to public health promotion and overall community well-being.

## 1. Introduction

Forest bathing is an outdoor meditative practice associated with benefits for the body and mind, including psychophysical relaxation, stress hormone reduction, and anxiety improvement [1,2,3]. Forest bathing sessions usually last two to three hours, and participants may be alone or accompanied by a therapist (forest therapy): the practice involves walking slowly in green areas along specific pathways, trying not to exert physical effort, but resting while contemplating the natural environment all around, and doing some light physical and breathing exercises in the forest [4]. For the sake of simplicity, both unsupervised and supervised experiences will be denoted as forest therapy in this article.

Despite originating in the Far East as a form of spiritual meditation to reconnect with nature, forest therapy has recently gained popularity in many Western countries, too, thus also becoming a trend in the field of well-being tourism [5]. As scientific evidence accumulates and more people become interested in the benefits of forest therapy, policy-makers have started to promote forest-based practices in different countries worldwide (including Japan, South Korea, New Zealand, the United Kingdom, and the European Union) in order to sustainably help address some socio-psychological issues and contribute to decreasing work-, study-, and chronic disease-related stress in the general population [4]. To date, most research studies about forest therapy have involved relatively small cohorts of healthy or sub-healthy adults [6,7], while little is known about the characteristics of forest therapy participants from the general population who engage in this practice on a voluntary basis, mostly driven by curiosity or recommended by a healthcare practitioner. In fact, in most of the primary studies available in the scientific literature, it can be observed that the sample was entirely made of individuals with homogeneous characteristics (for example, male university students, middle-aged females experiencing high levels of stress, patients with a specific health problem, etc.) [1,4,8]. It is worth noting that the characteristics of these study samples may not necessarily align with the diverse population that actively chooses to engage in forest therapy outside of scientific research settings. Given this disparity, it becomes crucial to gain an understanding of the demographics and psychosocial factors associated with individuals who willingly participate in forest therapy sessions. By examining the preferences and motivations of these individuals, researchers can study forest therapy from a more comprehensive psychosocial perspective. This approach allows for a deeper exploration of the broader population’s engagement with forest therapy and provides insights into the factors that attract people to this practice in real-world contexts. By expanding the research beyond homogeneous samples and encompassing a wider range of participants, it becomes possible to gather valuable information about the psychological, social, and cultural aspects of forest therapy. This knowledge can contribute to a more holistic understanding of the effects and benefits of forest therapy and guide the development of tailored interventions that cater to diverse populations. Ultimately, studying forest therapy from a psychosocial perspective helps bridge the gap between scientific research and the real-world experiences of individuals who choose to engage in this activity.

This research aims to describe the most important demographic, psychosocial, and lifestyle-related characteristics of participants in the largest forest therapy campaign ever carried out in Italy by public institutions. A secondary aim is to assess possible gender-based differences in these determinants.

## 2. Methods

### 2.1. Study Design and Participants

**Figure 1:**
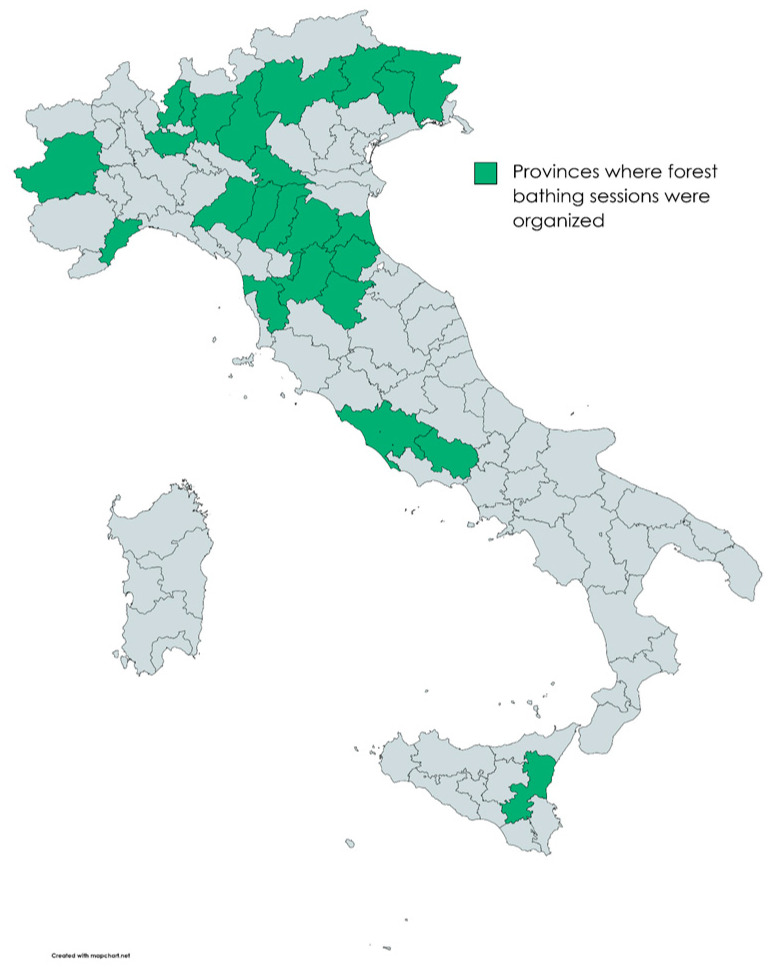
Italian provinces where forest therapy sessions were organized.

This study was designed as a multicenter cross-sectional survey [9]. A total of 1070 adults, who participated in standardized forest therapy experiences, were organized between June 2021 and October 2022 by the Italian Alpine Club (CAI—“Club Alpino Italiano”) and the National Research Council (CNR—“Consiglio Nazionale delle Ricerche”) of Italy, were surveyed. Forest therapy sessions were held in 39 forest sites across Italy, including mountain, hill, and urban park settings (Figure 1). Each site had previously been analyzed for specific structural characteristics, such as accessibility, safety, and moderate slope, ensuring the overall suitability for the experiences. 

The participants were recruited with a dedicated advertising campaign, including leaflet distribution and display of billboards in CAI headquarters, information dissemination through social media, and word-of-mouth promotion by private subjects. They all decided to participate in forest therapy sessions by themselves, without being referred to by doctors or other healthcare practitioners.

Forest therapy sessions were generally guided by mental health therapists (such as psychologists or psychotherapists), who accompanied the participants divided into groups of 15 to 20 people, gave standardized instructions to improve nature immersion, and made sure the people stayed safe during these outdoor experiences; in a few cases, billboards provided such instructions, while a mental health therapist made sure the experience was safe and comfortable. All mental health therapists who accompanied the participants had received standardized forest therapy training prior to conducting the experiences. An operator delegated by the CAI also attended the sessions to ensure the material safety of participants during forest therapy. 

Immediately before undertaking their forest therapy session (each lasting 2.5–3 h, without any time differences between guided and self-guided experiences), the participants were asked to fill in a paper-pencil survey with questions about their demographic and psychological characteristics. Only adults (aged 18+ years old) capable of providing their informed consent and with fluent Italian skills were considered eligible for the survey. All data collected in this way were fully anonymized and treated in accordance with current privacy regulations (“GDPR—Regolamento 2016/679”). On-site mental health therapists, along with CAI or CNR representatives, answered any doubts asked by the participants, who were free to withdraw from the survey at any time.

Following the survey, the participants engaged in a forest therapy session lasting approximately 2.5 to 3 h. The session involved leisurely walks of short duration, with frequent pauses at five different spots within the natural environment. At each stop, individuals were encouraged to unwind and immerse themselves in the surrounding forest scenery. The physical activity involved in the session was deliberately kept gentle and non-strenuous to avoid excessive activation of the adrenergic system. Participants were instructed to turn off their mobile phones and refrain from engaging in conversation with one another throughout the entire session, with the aim of fostering a profound connection with nature. It is important to note that forest therapy sessions were conducted only when the weather conditions were non-rainy, and the environmental temperature ranged between a comfortable 12 and 26 degrees Celsius.

### 2.2. Data Collection

The demographic characteristics collected in this survey were the participants’ age range, education level, occupational and relationship status, and living area (urban or rural). 

A questionnaire was specifically formulated in collaboration with the Section of Psychology of the University of Florence (Italy) to investigate the nature-related habits, awareness, and attitudes of forest therapy participants. This self-administered inventory was based on a 10-point Likert scale and consisted of five items, each ratable with a score ranging from 0 (most negative reply) to 10 (most positive reply). The overall score (also called “Nature Score”) was calculated by adding together all item scores and therefore ranged from 0 to 50. The five questions (translated into English) were the following:

1. How would you rate your psychophysical well-being when spending time in a green area?
  0—very poor; 10—very good
2. How familiar are you with forests?
  0—absolutely unfamiliar; 10—highly familiar
3. How important do you think your relationship with Nature is for who you are?
  0—not important at all; 10—very important
4. How strong do you think the connection between living beings and the Earth is?
  0—very weak connection; 10—very strong connection
5. How often do you think that your actions have an impact on the environment?
  0—never; 10—always

Along with the “Nature Score” questionnaire, the participants were asked how frequently they had recently spent some time in forests or parks (if more or less than twice a week).

Additionally, they were also administered the State-Trait Anxiety Inventory (STAI-T) [10], an internationally-validated psychometric questionnaire aimed to measure trait anxiety, which is part of an individual’s personality, in contrast with state anxiety, which is related to specific situations and conditions [11]. The levels of anxiety measured with the STAI questionnaire are defined as “no or low” (20–37), “moderate” (38–44), or “high” (45–80) depending on the participants’ STAI scores, as indicated in brackets [12]. 

Finally, lifestyle-related information was collected from the study participants, including their Body Mass Index (BMI), their smoking habits, whether they took medicinal drugs for chronic diseases, and their dietary habits (the number of servings of fruits and vegetables per day).

### 2.3. Statistical Analysis

After collection, the participants’ data were summarized in an Excel spreadsheet. The Shapiro–Wilk test was used to ensure that data distribution was normal. Continuous variables were presented as means and standard deviations, and gender-based differences (females versus males) were tested with the Student’s *t*-test. For dichotomous variables, data were presented as percentages of the total, and Fisher’s exact test was used to identify statistically significant gender-related differences. The threshold for statistical significance was conventionally set at *p* < 0.05. The statistical analysis was performed with “GraphPad QuickCalcs”, an online software freely accessible at: https://www.graphpad.com/ (access date: 1 April 2023).

## 3. Results

The main results of the survey are summarized in Table 1, Table 2, Table 3 and Table 4. The age range of the participants was mostly between 45 and 54 years old (86.7% of the study population). Females were 643, males 352, and those who preferred not to disclose their gender were 75. The education level of around half of the participants corresponded to a secondary school diploma (51.4%), while most of the remaining people had a university degree or completed a postgraduate course. Considering the occupational status, most people involved in forest therapy sessions were either employed or retired, while only a few were students or unemployed. Among those who were employed (n = 602), more than one-third (n = 249) had a so-called “green job”, namely an occupation that somehow contributes to environmental protection, nature preservation, and sustainable business activities [13]. More than 50% of the sample comprised people engaged in a marriage (most females were not married, while most males had a spouse). Around 60% of forest therapy participants were urban dwellers, while most remaining people were from rural areas (similar percentages applied for all gender subgroups).

With regard to nature-related habits, awareness, and attitudes, over 70% of forest therapy participants reported spending some time in green areas at least twice a week (Table 2). The percentage of female participants with this habit was superior to that of male subjects, but the difference was not statistically significant (F: 72.3% versus M: 68.2%). The average 0 to 50 Nature Score was quite high in the cohort surveyed (mean = 41.68 ± 7.21), and the number of respondents almost reached 90% of the study population, with limited dropouts. No significant difference was detected between female and male participants in terms of overall Nature Score. However, females reported significantly higher levels of well-being when spending time in green areas, and they declared to think that their actions have a greater impact on the environment than their male counterparts believed. Similarly, compared with responses provided by males, females reported thinking that living beings have a stronger connection with the Earth. On the contrary, male participants considered themselves significantly more familiar with forests.

**Table 2:** Nature-related habits, awareness, and attitudes of forest therapy participants (n = 1070).

		Overall (n = 1070)	Females (n = 643)	Males (n = 352)	ND (n = 75)
Frequency of visiting a forest or a park in the past 3 months	More than twice a week	70.4% (n = 753)	72.3% (n = 465)	68.2% (n = 240)	68.0% (n = 51)
	Gender-based difference (Females-Males)		Fisher’s exact test *p* = 0.19		
	Less than twice a week	28.1% (n = 301)	26.4% (n = 170)	30.7% (n = 108)	26.7% (n = 20)
	Gender-based difference (Females-Males)		Fisher’s exact test *p* = 0.16		
	ND	1.5% (n = 16)	1.2% (n = 8)	1.1% (n = 4)	5.3% (n = 4)
Nature Score 0–50 (mean ± SD)		41.68 ± 7.21 (n = 961)	41.86 ± 6.89 (n = 571)	41.96 ± 6.50 (n = 317)	39.07 ± 11.13 (n = 73)
% [n of participants who completed the survey]		89.8%	88.8%	90.1%	97.3%
Nature Score difference (Females-Males)			Δ = −0.10 [95% CI: −1.03; 0.83]; *p* = 0.83 t = 0.2094; df = 886; SE = 0.473		
Q1 Nature Score 0–10 (mean ± SD)		9.16 ± 1.09	9.25 ± 1.07	8.99 ± 1.11	9.01 ± 1.11
Q1 score difference (Females-Males)			Δ = 0.26 [95% CI: 0.12; 0.40]; *p* = 0.0004 * t = 3.5715; df = 983; SE = 0.072		
Q2 Nature Score 0–10 (mean ± SD)		7.77 ± 1.90	7.50 ± 2.00	8.26 ± 1.60	7.65 ± 1.91
Q2 score difference (Females-Males)			Δ = −0.77 [95% CI: −1.01; −0.52]; *p* = 0.0001 * t = 6.1802; df = 985; SE = 0.124		
Q3 Nature Score 0–10 (mean ± SD)		8.84 ± 1.42	8.83 ± 1.47	8.84 ± 1.33	8.66 ± 1.59
Q3 score difference (Females-Males)			Δ = −0.00 [95% CI: −0.19; 0.18]; *p* = 0.96 t = 0.0489; df = 983; SE = 0.095		
Q4 Nature Score 0–10 (mean ± SD)		8.21 ± 1.63	8.32 ± 1.61	8.01 ± 1.65	7.80 ± 2.12
Q4 score difference (Females-Males)			Δ = 0.30 [95% CI: 0.09; 0.52]; *p* = 0.005 * t = 2.8141; df = 984; SE = 0.108		
Q5 Nature Score 0–10 (mean ± SD)		8.46 ± 1.51	8.58 ± 1.45	8.23 ± 1.60	8.20 ± 1.44
Q5 score difference (Females-Males)			Δ = 0.35 [95% CI: 0.16; 0.55]; *p* = 0.0005 * t = 3.5175; df = 983; SE = 0.100		

Legends. ND = Not Disclosed. The *t*-test was used to calculate the difference between the mean values of gender subgroups. * Statistically significant difference (*p* < 0.05).

The STAI-T scores indicated that, on average, forest therapy participants had moderate levels of trait anxiety (Table 3). This tendency was more pronounced among the women, with a significant difference between females and males. The number of STAI questionnaires returned by the survey participants was around 86%. 

**Table 3:** STAI-T scores of forest therapy participants.

	Overall (n = 1070)	Females (n = 643)	Males (n = 352)	ND (n = 75)
STAI-T score (mean ± SD)	42.27 ± 9.61 (n = 925)	43.63 ± 9.67 (n = 555)	39.81 ± 9.29 (n = 306)	42.25 ± 8.23 (n = 64)
% [participants who completed the survey]	86.4%	86.3%	86.9%	85.3%
STAI-T score difference (Females-Males)		Δ = 3.82 [95% CI: 2.48; 5.15]; *p* = 0.0001 * t = 5.6237; df = 859; SE = 0.679		

Legends. ND = Not Disclosed. The *t*-test was used to calculate the difference between the mean values of gender subgroups. All questionnaires were administered immediately before taking part in a forest therapy session. * Statistically significant difference (*p* < 0.05).

The general lifestyle-related information of the study participants is summarized in Table 4. The average BMI for the entire sample was 23.6 kg/m^2^, with a statistically significant difference observed between females and males. Female participants, on average, had a lower BMI than their male counterparts. A relatively small proportion of the study subjects reported being smokers (13.8%), with no significant differences based on gender. Approximately half of the study sample consisted of individuals who took at least one medicinal drug daily for chronic disease, and there were no significant differences between males and females in terms of regular medication use. More than half of the study participants consumed a minimum of three servings of fruits and vegetables per day. However, there were significant differences in dietary habits between males and females, as indicated in Table 4.

**Table 4:** Lifestyle-related characteristics of forest therapy participants (n = 1070).

		Overall (n = 1070)	Females (n = 643)	Males (n = 352)	ND (n = 75)
Body Mass Index (BMI) [kg/m^2^]	Mean ± SD (n)	23.6 ± 3.5 (n = 1046)	22.7 ± 3.3 (n = 632)	25.3 ± 3.4 (n = 345)	24.0 ± 3.4 (n = 69)
	BMI difference (Females-Males)		Δ = −2.52 [95% CI: 2.96; 2.08]; *p* < 0.0001 * t = 11.3494; df = 975; SE = 0.222		
Smoking habit	Yes (%, n)	13.8% (n = 145/1054)	13.5% (n = 86/635)	12.0% (n = 42/349)	24.3% (n = 17/70)
	Difference in smoking habit (Females-Males)		Fisher’s exact test *p* = 0.55		
Medicinal drugs taken for chronic diseases	Yes, at least a drug every day (%, n)	41.6% (n = 439/1056)	42.7% (n = 272/637)	40.4% (n = 141/349)	37.1% (n = 26/70)
	Difference in medicinal drugs (Females-Males)		Fisher’s exact test *p* = 0.50		
Daily consumption of vegetables and fruits (%, n)	Less than 2 servings	43.8% (n = 469)	39.7% (n = 255)	51.4% (n = 181)	44.0% (n = 33)
	Difference in dietary habits (Females-Males)		Fisher’s exact test *p* = 0.0004 *		
	3–5 servings	48.4% (n = 518)	51.8% (n = 333)	43.5% (n = 153)	42.7% (n = 32)
	Difference in dietary habits (Females-Males)		Fisher’s exact test *p* = 0.0141 *		
	More than 5 servings	6.2% (n = 66)	7.6% (n = 49)	3.7% (n = 13)	5.3% (n = 4)
	Difference in dietary habits (Females-Males)		Fisher’s exact test *p* = 0.0029 *		
	ND	1.6% (n = 17)	0.9% (n = 6)	1.4% (n = 5)	8.0% (n = 6)

Legends. ND = Not Disclosed. The results are expressed as percentages of the total and units. The *t*-test or Fisher’s exact test was used to calculate the difference between the mean values of gender subgroups. * Statistically significant difference (*p* < 0.05).

Out of the total sessions, there were four self-guided sessions and 51 guided walks (some sites were used more than once), which means that self-guided sessions accounted for 7.3% of all sessions (7.2% of participants). It is worth noting that every self-guided session was always paired with a guided session, and participants were unaware in advance of which session they were assigned to. The allocation was completely random and based on extracted codes, ensuring that participation was not influenced by the type of session when answering the survey. Since every self-guided session was always accompanied by a guided session, there was no correlation with natural features. The four self-guided sessions took place in the provinces of Pisa, Tuscany (hilly site); Mantova, Lombardy (urban park); Trento (mountain site); and Pordenone, Friuli (mountain site). Among participants in different sessions with at least 15 individuals, there were no significant gender differences. The most frequently visited sites (above the 70th percentile) were either easily accessible or located near densely populated areas. These included a hilly site in the province of Pisa, an urban park in Rome, a historic pinewood in the province of Ravenna, the forest surrounding the well-known spa of Fiuggi in the province of Frosinone, a low mountain site near Florence, and an alpine site not far from Milan. There were notable exceptions, such as an enchanting alpine refuge in the Susa Valley, province of Torino, characterized by an extensive larch forest with creeks and small lakes; a completely natural alpine valley with stunning vegetational biodiversity in the province of Pordenone, located in the eastern Italian Alps; the attractive and highly frequented Tovel lake in the province of Trento, with an indigenous and extensive spruce forest; the largest beech forest in the Apennines, province of Reggio Emilia; and two sites around the incredibly scenic Etna volcano. Apart from the easily accessible sites, it seems that the most visited locations were characterized by more natural and protected forest types, as well as highly appealing landscapes, compared to all other sites.

## 4. Discussion

The majority of forest therapy participants fell into the age range of their 40s or 50s, aligning with the average demographic profile of the Italian population (refer to Table 5). Notably, there was a significant imbalance in gender representation, with a considerably higher number of female participants than males, suggesting that forest therapy holds greater appeal for women. It is worth mentioning that although women comprise less than 40% of the overall Italian population (as indicated in Table 5), they formed the majority in our study sample. This observation underscores the popularity of forest therapy among women. Interestingly, the study sample was predominantly composed of working individuals, consistent with the fact that forest therapy has recently emerged as a proposed solution to alleviate occupational stress levels [14]. It is important to note that while workers constituted the majority of participants, this might not necessarily reflect the proportional distribution in the general Italian population (as detailed in Table 5).

Furthermore, a significant number of participants resided in urban areas. However, it is worth highlighting that rural dwellers were overrepresented in our study sample when compared to national data. This is notable considering that less than 30% of the Italian population lives in the countryside, as reported in Table 5. This finding emphasizes the need for policy-makers in Italy to promote and encourage urban residents to engage more frequently in outdoor activities within forests or parks. Living in urban areas with limited access to green spaces has been associated with heightened levels of stress and diminished quality of life [15].

In terms of educational background, it was observed that forest therapy participants exhibited a higher proportion of individuals with a university degree or postgraduate education, accounting for over 45% of the study sample. This indicates a preference among individuals with higher levels of education to engage in forest therapy and participate in related research. It is crucial to make additional efforts to communicate the benefits of forest therapy and disseminate scientific knowledge among those with a lower level of education in order to ensure inclusivity and reach a broader audience. Notably, it should be mentioned that the percentage of individuals with a university degree or postgraduate education in Italy does not surpass one-third of the entire population, as indicated in Table 5.

**Table 5:** Demographic characteristics of the Italian population (2021).

	Mean Age	Gender	Graduated	Employed	Married	Urban
Italian population	45.9 years old (43.3–49.2 depending on the region)	N of males: ~29 million N of females: ~30 million	8.1–27.8% depending on the age range	22.55 million (38.1% of the overall population)	48.3% of men and 46.3% of women (2019)	71.35% of the overall population

Data are derived from the open access database of “Statista”, found online at https://www.statista.com/ (access date: 3 April 2023).

As evident from the findings of this survey and outlined in Table 2, most individuals who opted for forest therapy already engage in regular visits to green areas, spending at least two days a week in such environments. Moreover, they demonstrate a commendable level of awareness regarding fundamental environmental concepts. These concepts include recognizing the advantages of outdoor activities in a forest setting, understanding the interconnectedness between our well-being and the well-being of the Earth, and comprehending the impact of personal actions on the natural environment. To further enhance environmental awareness among those less acquainted with these concepts, it is crucial to involve them in outdoor activities that not only offer recreation and promote health but also serve educational purposes. For instance, to attract a wider audience and increase environmental consciousness, billboards or images briefly illustrating pertinent environmental issues could be strategically placed along select forest trails or at the entrances. This approach could be integrated following the promotion of forest therapy for its well-being benefits. By combining recreational experiences with educational components, individuals can be encouraged to develop a deeper understanding of environmental topics and their significance.

Concerning lifestyle-related information, the survey showed that forest therapy participants tended to have quite healthy habits, and their average BMI was within the interval for normality (19–25 kg/m^2^) [16], despite being close to the upper threshold. However, compared with females, males were overweight on average, with a mean BMI above 25 kg/m^2^ (Table 4). Forest therapy sessions may be an occasion to promote outdoor physical activity and indirectly help these subjects better control their body weight. The percentage of smokers among the participants was markedly lower than that reported for the Italian population (13.8% of the study sample versus 24.2% of the national population) [17]. Around 55% of the survey participants reported eating three or more servings of fruits and vegetables daily. Even though the World Health Organization recommends consuming at least five servings of fruits and vegetables [18], recent studies suggest that three-to-four servings are sufficient to significantly reduce the risk of cardiovascular and noncardiovascular diseases [19]. Our survey showed that, among forest therapy participants, a higher percentage of males tended to consume less than three servings of fruits and vegetables. In the future, forest therapy sessions may be followed or accompanied by information campaigns about healthy nutrition aimed at improving the participants’ dietary habits, especially those of males.

The number of subjects taking at least a medicinal drug every day for chronic disease was less than half of the entire study sample. In this regard, it would be important to understand whether the regular practice of forest therapy can help these patients better cope with their health conditions. Other studies have already explored the potential integrative role of forest therapy for improving different psychophysical diseases [4]; in particular, in addition to showing for the first time the specific anxiolytic effect of monoterpenes emitted by plants and available in the forest atmosphere, recent research has hypothesized indirect benefits for cardiovascular health promotion among participants to forest therapy experiences [3]. Future trials should address these issues in more depth and define whether and how forest therapy can be integrated into multidisciplinary public health programs contributing to primary or secondary disease prevention.

Italian research has recently begun to delve into the potential benefits of forest therapy. Most of the studies conducted thus far have focused on specific groups of healthy or sub-healthy adults to assess the effectiveness of this practice in enhancing psychophysical well-being [3,20,21]. Findings from larger studies conducted in other countries have indicated that, in general, females tend to derive greater psychological benefits from forest therapy compared to males [2,22]. This observation may help explain the higher participation rate of women in this outdoor activity within Italy. Furthermore, epidemiological surveys exploring the utilization of complementary and integrative therapies in Italy have revealed that individuals who seek such therapeutic practices, including mind–body interventions, acupuncture, herbal medicine, and homeopathy, share similar characteristics with forest therapy participants. Specifically, these individuals are predominantly highly educated women in their 30s or 40s [23]. These findings align with the primary outcomes of our study, which further supports the notion that as evidence continues to accumulate regarding its beneficial effects, forest therapy has been increasingly proposed as a complementary practice for healthcare and the promotion of overall well-being [24].

### 4.1. Study Limitations

This study was an observational survey based on self-report questionnaires, and the data collected in this way can be subject to different types of bias, including social desirability response and reporting biases [25,26]. In order to reduce the impact of these sources of data distortion on the participant’s responses, the study sample was kept as large as possible, and the mental health therapists who accompanied the people during forest therapy sessions helped those who had doubts or needed some help to fill in the inventories. This survey was conducted in Italy, and its results are not directly generalizable worldwide, as cultural, psychosocial, and demographic differences can have an impact on the overall findings. Therefore, in other countries, the average characteristics of the majority of forest therapy participants may be different.

### 4.2. Practical Implications

This study has practical implications in various areas. Firstly, considering the existing gender gap in happiness and well-being, forest therapy and other outdoor activities that foster a connection with nature could be utilized to promote psychological well-being, specifically among women [27]. By engaging in such activities, women may experience positive effects on their mental health. 

Secondly, the study observed that most men participating in forest therapy sessions were overweight on average. To address this, tailored interventions such as providing nutritional advice or incorporating more dynamic sessions could be proposed for male subgroups. This approach could help improve the physical well-being of male participants and encourage healthier lifestyles.

Thirdly, in Italy, forest therapy was found to have a limited impact on raising environmental awareness. It was noted that individuals who already have an interest in forest therapy tend to be conscious of important environmental issues. Therefore, additional efforts should be directed toward targeting the wider population and increasing awareness through alternative initiatives or programs.

Fourthly, while forest therapy typically does not involve strenuous physical activity, it does incorporate some physical exercises. Given that a significant proportion of forest therapy participants have chronic diseases that require pharmacological treatment, seeking medical advice before engaging in outdoor activities in natural green areas is crucial. This ensures that individuals with pre-existing health conditions receive appropriate guidance to ensure their safety and well-being during the sessions.

In summary, the practical implications of this study include promoting psychological well-being among women, offering tailored interventions for overweight male participants, targeting a broader population for environmental awareness, and emphasizing the importance of medical advice for individuals with chronic diseases participating in forest therapy.

## 5. Conclusions

In Italy, forest therapy participants typically exhibit several common characteristics. Firstly, the majority of participants are female, falling within the age range from 45 to 54 years old. They are generally employed individuals who are not married. Moreover, forest therapy participants in Italy tend to possess a higher level of education, often residing in urban areas. These individuals display a commendable level of environmental awareness and maintain a nature-oriented attitude. Additionally, they tend to have moderate levels of trait anxiety. Furthermore, forest therapy participants in Italy are more likely to be nonsmokers, maintain an average normal body mass index (BMI), and consume an adequate daily amount of fruits and vegetables. It is important to note that their male counterparts, on the other hand, often struggle with overweight issues and exhibit poorer dietary habits. Exploring these gender disparities and addressing them within the context of forest therapy can be a valuable area for future investigation. To expand our understanding, it would be beneficial for future studies to examine whether these characteristic patterns persist across different countries. Additionally, exploring the effectiveness of health-promoting interventions integrated with forest therapy sessions in addressing these issues among forest therapy participants can enhance public health and community well-being.

## Figures and Tables

**Table 1:** Demographic characteristics of forest therapy participants (n = 1070).

		Overall (n = 1070)	Females (n = 643)	Males (n = 352)	ND (n = 75)
Age range (years)	18–29	0.8% (n = 9)	1.1% (n = 7)	0.6% (n = 2)	0.0% (n = 0)
	30–44	2.7% (n = 29)	3.0% (n = 19)	2.3% (n = 8)	2.7% (n = 2)
	45–54	86.7% (n = 928)	87.1% (n = 560)	85.8% (n = 302)	88.0% (n = 66)
	55–69	8.0% (n = 86)	7.5% (n = 48)	9.1% (n = 32)	8.0% (n = 6)
	70+	1.7% (n = 18)	1.4% (n = 9)	2.3% (n = 8)	1.3% (n = 1)
Education level	Primary school	1.6% (n = 17)	0.8% (n = 5)	2.8% (n = 10)	2.7% (n = 2)
	Secondary school	51.4% (n = 550)	48.5% (n = 312)	55.7% (n = 196)	56.0% (n = 42)
	University degree	34.8% (n = 372)	37.5% (n = 241)	31.5% (n = 111)	26.7% (n = 20)
	Postgraduate education	10.9% (n = 117)	12.3% (n = 79)	9.4% (n = 33)	6.7% (n = 5)
	ND	1.3% (n = 14)	0.9% (n = 6)	0.6% (n = 2)	8.0% (n = 6)
Occupational status	Employed	56.3% (n = 602)	58.5% (n = 376)	52.6% (n = 185)	54.7% (n = 41)
	Student	2.6% (n = 28)	2.8% (n = 18)	2.6% (n = 9)	1.3% (n = 1)
	Unemployed	6.2% (n = 66)	7.8% (n = 50)	3.4% (n = 12)	5.3% (n = 4)
	Retired	32.1% (n = 344)	28.1% (n = 181)	39.2% (n = 138)	33.3% (n = 25)
	ND	2.8% (n = 30)	2.8% (n = 18)	2.3% (n = 8)	5.3% (n = 4)
Relationship status	Married	51.6% (n = 552)	46.3% (n = 298)	60.8% (n = 214)	52.0% (n = 39)
	Not married	46.5% (n = 498)	52.1% (n = 335)	38.4% (n = 135)	38.7% (n = 29)
	ND	1.9% (n = 20)	1.6% (n = 10)	0.9% (n = 3)	9.3% (n = 7)
Living area	Urban	59.2% (n = 633)	58.7% (n = 377)	59.4% (n = 209)	62.7% (n = 47)
	Rural	39.8% (n = 426)	40.4% (n = 260)	40.3% (n = 142)	32.0% (n = 24)
	ND	1.0% (n = 11)	0.9% (n = 6)	0.3% (n = 1)	5.3% (n = 4)

Legends. ND = Not Disclosed. The results are expressed as percentages of the total and units.

## Data Availability

All data are available by contacting the corresponding author.

## References

[1] Park B.J., Tsunetsugu Y., Kasetani T., Kagawa T., Miyazaki Y. (2010). The Physiological Effects of Shinrin-Yoku (taking in the Forest Atmosphere or Forest Bathing): Evidence from Field Experiments in 24 Forests across Japan. Environ. Health Prev. Med..

[2] Kotera Y., Richardson M., Sheffield D. (2022). Effects of Shinrin-Yoku (forest Bathing) and Nature Therapy on Mental Health: A Systematic Review and Meta-Analysis. Int. J. Ment. Health Addict..

[3] Donelli D., Meneguzzo F., Antonelli M., Ardissino D., Niccoli G., Gronchi G., Baraldi R., Neri L., Zabini F. (2023). Effects of Plant-Emitted Monoterpenes on Anxiety Symptoms: A Propensity-Matched Observational Cohort Study. Int. J. Environ. Res. Public Health.

[4] Antonelli M., Donelli D., Carlone L., Maggini V., Firenzuoli F., Bedeschi E. (2022). Effects of Forest Bathing (shinrin-Yoku) on Individual Well-Being: An Umbrella Review. Int. J. Environ. Health Res..

[5] Farkic J., Isailovic G., Taylor S. (2021). Forest Bathing as a Mindful Tourism Practice. Ann. Tour. Res. Empir. Insights.

[6] Wen Y., Yan Q., Pan Y., Gu X., Liu Y. (2019). Medical Empirical Research on Forest Bathing (Shinrin-Yoku): A Systematic Review. Environ. Health Prev. Med..

[7] Tsunetsugu Y., Park B.-J., Miyazaki Y. (2010). Trends in Research Related to “Shinrin-Yoku” (taking in the Forest Atmosphere or Forest Bathing) in Japan. Environ. Health Prev. Med..

[8] Hansen M.M., Jones R., Tocchini K. (2017). Shinrin-Yoku (Forest Bathing) and Nature Therapy: A State-of-the-Art Review. Int. J. Environ. Res. Public Health.

[9] Sharma A., Minh Duc N.T., Luu Lam Thang T., Nam N.H., Ng S.J., Abbas K.S., Huy N.T., Marušić A., Paul C.L., Kwok J. (2021). A Consensus-Based Checklist for Reporting of Survey Studies (CROSS). J. Gen. Intern. Med..

[10] Spielberger C.D. (2010). State-Trait Anxiety Inventory. Corsini Encyclopedia of Psychology.

[11] Reiss S. (1997). Trait Anxiety: It’s Not What You Think It Is. J. Anxiety Disord..

[12] Kayikcioglu O., Bilgin S., Seymenoglu G., Deveci A. (2017). State and Trait Anxiety Scores of Patients Receiving Intravitreal Injections. Biomed. Hub.

[13] Stanef-Puică M.-R., Badea L., Șerban-Oprescu G.-L., Șerban-Oprescu A.-T., Frâncu L.-G., Crețu A. (2022). Green Jobs-A Literature Review. Int. J. Environ. Res. Public Health.

[14] Furuyashiki A., Tabuchi K., Norikoshi K., Kobayashi T., Oriyama S. (2019). A Comparative Study of the Physiological and Psychological Effects of Forest Bathing (Shinrin-Yoku) on Working Age People with and without Depressive Tendencies. Environ. Health Prev. Med..

[15] Roe J.J., Thompson C.W., Aspinall P.A., Brewer M.J., Duff E.I., Miller D., Mitchell R., Clow A. (2013). Green Space and Stress: Evidence from Cortisol Measures in Deprived Urban Communities. Int. J. Environ. Res. Public Health.

[16] Website N.H.S. What Is the Body Mass Index (BMI)?. https://www.nhs.uk/common-health-questions/lifestyle/what-is-the-body-mass-index-bmi/.

[17] Press Release N°39/2022—Smoking: Italy Reports Almost 800,000 Smokers More than in 2019 and the Consumption of Heated Tobacco Products Has Tripled. https://www.iss.it/-/no-tobacco-day-2022-iss-en.

[18] Healthy Diet. https://www.who.int/news-room/fact-sheets/detail/healthy-diet.

[19] Miller V., Mente A., Dehghan M., Rangarajan S., Zhang X., Swaminathan S., Dagenais G., Gupta R., Mohan V., Lear S. (2017). Fruit, Vegetable, and Legume Intake, and Cardiovascular Disease and Deaths in 18 Countries (PURE): A Prospective Cohort Study. Lancet.

[20] Borriello G., Grazioli E., Zavarella P., Paolini R., Ianniello A., Silvestri D., Cerulli C., Parisi A. (2022). Experiencing Forest Therapy in the Italian Landscape: Bathing in the Selva of Castelfidardo. Preprints.org.

[21] Meneguzzo F., Albanese L., Antonelli M., Baraldi R., Becheri F.R., Centritto F., Donelli D., Finelli F., Firenzuoli F., Margheritini G. (2021). Short-Term Effects of Forest Therapy on Mood States: A Pilot Study. Int. J. Environ. Res. Public Health.

[22] Subirana-Malaret M., Miró A., Camacho A., Gesse A., McEwan K. (2023). A Multi-Country Study Assessing the Mechanisms of Natural Elements and Sociodemographics behind the Impact of Forest Bathing on Well-Being. For. Trees Livelihoods.

[23] Menniti-Ippolito F., Gargiulo L., Bologna E., Forcella E., Raschetti R. (2002). Use of Unconventional Medicine in Italy: A Nation-Wide Survey. Eur. J. Clin. Pharmacol..

[24] Kotte D., Li Q., Shin W.S., Michalsen A. (2019). International Handbook of Forest Therapy.

[25] Chan D., Lance C.E. (2009). So Why Ask Me? Are Self-Report Data Really That Bad?. Statistical and Methodological Myths and Urban Legends: Doctrine, Verity and Fable in the Organizational and Social Sciences.

[26] Donaldson S.I., Grant-Vallone E.J. (2002). Understanding Self-Report Bias in Organizational Behavior Research. J. Bus. Psychol..

[27] Rizzato M., Antonelli M., Sam C., Di Dio C., Lazzeroni D., Donelli D. (2023). Happiness and Socio-Demographic Factors in an Italian Sample: A Propensity-Matched Study. Healthc. Pap..

